# Sequential Organ Failure Assessment Score for Evaluating Organ Failure and Outcome of Severe Maternal Morbidity in Obstetric Intensive Care

**DOI:** 10.1100/2012/172145

**Published:** 2012-02-14

**Authors:** Antonio Oliveira-Neto, Mary A. Parpinelli, Jose G. Cecatti, Joao P. Souza, Maria H. Sousa

**Affiliations:** ^1^Intensive Care Unit, Department of Obstetrics and Gynecology, School of Medical Sciences, University of Campinas, Campinas, SP, Brazil; ^2^Obstetric Unit, Department of Obstetrics and Gynecology, School of Medical Sciences, University of Campinas, Campinas, SP, Brazil; ^3^Center of Studies on Reproductive Health of Campinas, Campinas, SP, Brazil

## Abstract

*Objective*. To evaluate the performance of Sequential Organ Failure Assessment (SOFA) score in cases of severe maternal morbidity (SMM). *Design*. Retrospective study of diagnostic validation. *Setting*. An obstetric intensive care unit (ICU) in Brazil. *Population*. 673 women with SMM. Main Outcome Measures. mortality and SOFA score. *Methods*. Organ failure was evaluated according to maximum score for each one of its six components. The total maximum SOFA score was calculated using the poorest result of each component, reflecting the maximum degree of alteration in systemic organ function. *Results*. highest total maximum SOFA score was associated with mortality, 12.06 ± 5.47 for women who died and 1.87 ± 2.56 for survivors. There was also a significant correlation between the number of failing organs and maternal mortality, ranging from 0.2% (no failure) to 85.7% (≥3 organs). Analysis of the area under the receiver operating characteristic (ROC) curve (AUC) confirmed the excellent performance of total maximum SOFA score for cases of SMM (AUC = 0.958). *Conclusions*. Total maximum SOFA score proved to be an effective tool for evaluating severity and estimating prognosis in cases of SMM. Maximum SOFA score may be used to conceptually define and stratify the degree of severity in cases of SMM.

## 1. Introduction

Potentially severe complications are estimated to occur in 15% of pregnancies, resulting in 529.000 maternal deaths annually worldwide [1]. Maternal deaths arise from the risk attributable to pregnancy as well as from the poor-quality care from health services [2]. Ensuring equitable access to basic and emergency skilled care, the early recognition and treatment of maternal potentially life-threatening conditions is critical for saving the lives of mothers and their newborns [1, 3].

Maternal death should be understood as the final stage of an ongoing condition of severe maternal morbidity (SMM) [4, 5]. Organ dysfunction is a continuous, dynamic process of alterations in organ function [6, 7] and is part of the pathophysiologic process of SMM [7–10]. Different patient populations develop different patterns of organ dysfunction [11]. Studies that have evaluated SMM in intensive care units (ICU) have reported that the degree of organ dysfunction, the number of failing organs, and the duration of the condition were variables directly related to higher maternal mortality [8, 9, 12–14].

With the objective of stratifying severity, evaluating therapeutic response, and estimating prognoses in cases of SMM, scores traditionally used in ICUs, such as the Acute Physiology and Chronic Health Evaluation II (APACHE II), Mortality Probability Models (MPMs), and the Simplified Acute Physiology Score II (SAPS II) [15–17], have been applied to this patient population with conflicting results, generally leading to overestimation of the severity of illness and maternal mortality [8, 13, 18–23]. Of the methods proposed, those evaluating organ dysfunction appear to offer greater sensitivity and specificity [9].

The Sequential Organ Failure Assessment (SOFA) score [7] was developed and later validated as a tool for quantifying the degree of organ dysfunction and the prognosis of severely ill patients [24–27]. Total maximum SOFA score, a measurement resulting from and complementing the SOFA score, takes into consideration the maximum degree of alteration in organ function resulting from the insult suffered during the period the patient remained in the ICU [28].

The patterns of occurrence of organ dysfunction in women with severe complications of pregnancy, and the evolution of these patients, constitute a subject that has so far received sparse attention. The objective of this study was to evaluate organ dysfunction and failure and the discriminatory power (the ability of the method to distinguish between non-survivors and survivors) of total maximum SOFA score in cases of SMM admitted to an obstetric ICU. The long-term objective of this approach is to provide evidence for supporting the decision of using organ dysfunction and failure as the criteria for defining maternal near miss.

## 2. Methods

This retrospective study was carried out at the Center for Women's Integrated Healthcare (CAISM) in Campinas, São Paulo, Brazil. CAISM is a public teaching hospital and is part of the hospital complex of the University of Campinas in which around 2,900 deliveries are performed annually. This hospital, which serves as a tertiary referral center for a catchment area with a population of around three million, is equipped with an ICU designed to provide intensive monitoring and physiological support for women with multiorgan failure and/or requiring prolonged assisted ventilation.

The multidisciplinary team responsible for patient care is composed of an intensive care physician, a resident obstetrician, a nurse, three auxiliary nurses, and a respiratory physiotherapist in addition to a senior obstetrician 24 hours on-site, and specialized surgeons are available whenever required. The ICU is an open unit (receiving cases from the same hospital or from outside) with 6 beds, with early involvement integrating all members of the multidisciplinary team. The clinical complications are managed by ICU team, but the obstetric staff is also closely involved in managing patients. The obstetric team is responsible for surgical interventions, regular fetal heart monitoring, indicating in some cases of severe maternal illness the better option for treatment, mode, and time of delivery.

This obstetric ICU receives annually around 140 cases of SMM, and the criteria for admission to this unit follow the guidelines of the American College of Critical Care Medicine and the Society of Critical Care [29] and are in compliance with the criteria proposed for obstetrical ICUs [21, 30–34]. In the present study, all the obstetrical admissions that occurred from August 2002 until September 2007 were analyzed. The data were collected from the medical records by two of the investigators and two research assistants. A protocol for the abstraction of the data was developed, tested, and reviewed by the team prior to initiation of the study.

For each admission to the ICU, data were collected retrospectively on variables that included the characteristics of the women, the reason for their admission to the ICU and the treatment they received there, any procedures or interventions constituting advanced life support, and the variables used for calculation of the SOFA score [6]. Further details on the methods used in the abstraction of the data and characterization of the population may be found in another publication [35]. This study was only implemented following approval of the proposed protocol by the local Institutional Review Board.

Total maximum SOFA score was used to evaluate and define organ dysfunction/failure during the entire time the patient remained in the ICU. Maximum SOFA score (0–4 points) is determined for each one of the six components, using the poorest result of each variable recorded during the entire period the patient remained in the ICU. For the purposes of analysis, organ dysfunction was defined as a maximum SOFA score ≥1 and ≤2, while a score of ≥3 points was considered organ failure. The aggregated result represents the total maximum SOFA score (0–24 points) and reflects the maximum degree of alteration in systemic organ function. The score was calculated in accordance with the original publication [28]. Thus, the maximum score was not a snapshot of the patient's condition on one particular day but a score that can only be calculated in retrospect. Therefore, in this way it has a limited relevance for clinical management. In fact it was studied to test the hypothesis that organ dysfunction and failure were highly correlated to mortality and severe morbidity in order to support the adoption of these parameters for defining criteria for maternal near miss.

Since arterial blood gas analysis has not been routinely recorded in all women admitted to the ICU but performed whenever required and at the criteria of the intensive care physician, records of partial pressure of oxygen (PaO_2_) were not available in all cases. In some cases, evaluation of the neurological system according to the Glasgow coma score was impossible due to the use of continuous sedation. In these very few situations, unless there was any sign to the contrary, the parameter was assumed to be normal and the normal value for the variable was used in calculating the score [26].

## 3. Statistical Analysis

Initially, a bivariate analysis was carried out to describe the mean SOFA score and standard deviation for each organ or system evaluated, according to the outcome of ICU admission (death or survival) by applying the Mann-Whitney nonparametric test for independent samples. Next, graphs were constructed containing the number of women with SMM, the rate of deaths per category, and the regression adjustments of the death rates according to the total maximum SOFA score, the number of failing organs and, later, according to each system evaluated individually. For each system assessed, sensitivity, specificity, and the area under the curve (AUC) were evaluated, together with their respective 95% confidence intervals (95% CI). Next, the ROC curve was fitted, and simultaneous confidence bands were plotted considering maximum likelihood estimates for the total maximum SOFA score, assuming a binormal distribution [36], and empirical values of sensitivity and specificity were presented. Finally, a logistic regression model was adjusted according to the outcome of admission to the ICU (death) considering total maximum SOFA score as the only independent variable, and the estimated probability of death was calculated for each case. Significance level was defined at 5%, and the software programs used were the Statistical Package for the Social Sciences (SPSS) for Windows, version 11.5 and Stata, version 7.0.

## 4. Results

During the period studied, 673 cases of SMM were admitted to the ICU, while over the same period, 14,440 deliveries were performed in the hospital, resulting in an admission rate to the ICU of 4.6%. Obstetrical complications were responsible for 66.5% of admissions. During the study period, 18 maternal deaths occurred, representing 2.6% of admissions to the ICU. Further details of the demographic characteristics of the study population are available in a previous publication [35]. 

**Figure 1:**
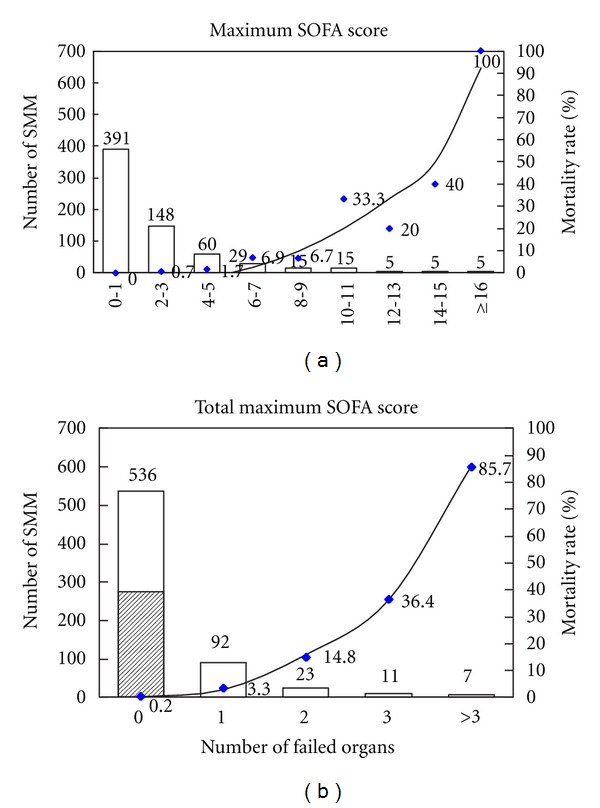
Number of severe maternal morbidity (SMM) patients (bar), mortality rate (symbol), and regression adjustment of mortality, according to total maximum Sequential Organ Failure Assessment (SOFA) score (a) and number of failed organs (b). Hatched area represents 273 cases of organ dysfunction (b).

Total maximum SOFA score was significantly higher in the patients who died compared to survivors (Table 1). A regression curve for mortality showed a substantial increase in the mortality rate as a function of total maximum SOFA score (Figure 1(a)). Organ dysfunction and/or failure diagnosed according to maximum SOFA score was found in 61.1% of admissions to the ICU, with dysfunction occurring in 273 women (40.6%) and failure of one or more organs in 138 women, representing one-fifth of cases of SMM. A significant correlation was also found between the number of failed organs and mortality, with mortality varying from 0.2% in women with no organ failure to 85.7% in those in whom failure of three or more organs occurred (Figure 1(b)). There was only one patient who died with no organ failure according to the maximum SOFA score.

**Figure 2:**
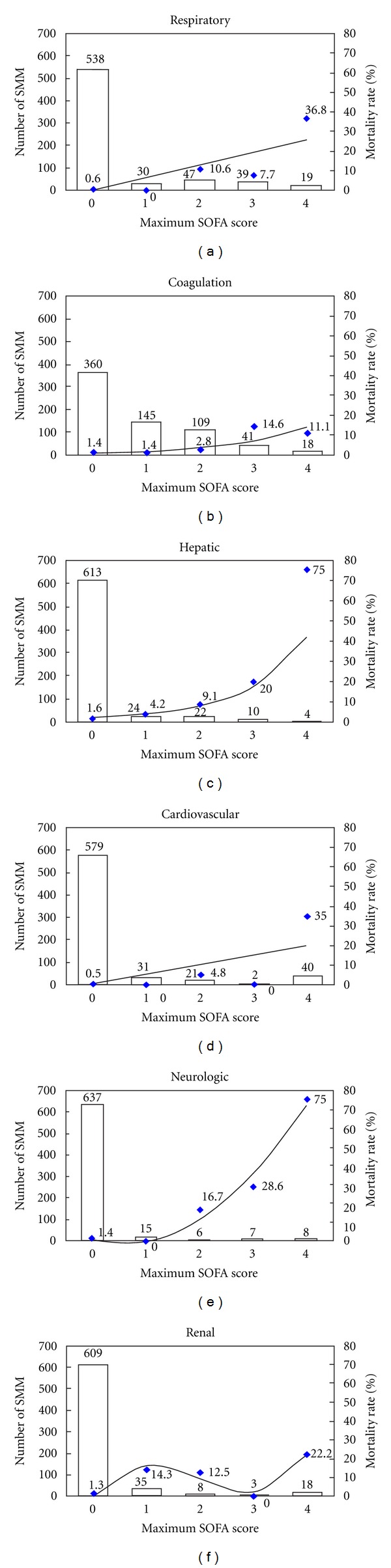
Number of Severe Maternal Morbidity (SMM) patients (bar), mortality rate (symbol) and regression adjustment of mortality, according to maximum Sequential Organ Failure Assessment (SOFA) score for each system evaluated.

The estimated probability of death for this obstetrical population was calculated for each individual value in the maximum total SOFA score and is shown in Table 2. Analysis of organ function according to maximum SOFA score showed a significant correlation between mortality and a higher score. The ratio between the number of cases in each category of maximum SOFA score (0–4) and the corresponding mortality rate is shown in Figure 2. Mortality increased as a function of maximum SOFA score, mainly with respect to the scores for hepatic and neurological function. For the scores referring to respiratory, coagulation and cardiovascular function, this trend was less strong, while in the case of renal function, no correlation was found. The number of cases of organ failure in the six components of the total maximum SOFA score ranged, in decreasing order, from coagulation (59), respiratory (58), cardiovascular (42), renal (21), and neurological (16) to hepatic (14).

**Figure 3:**
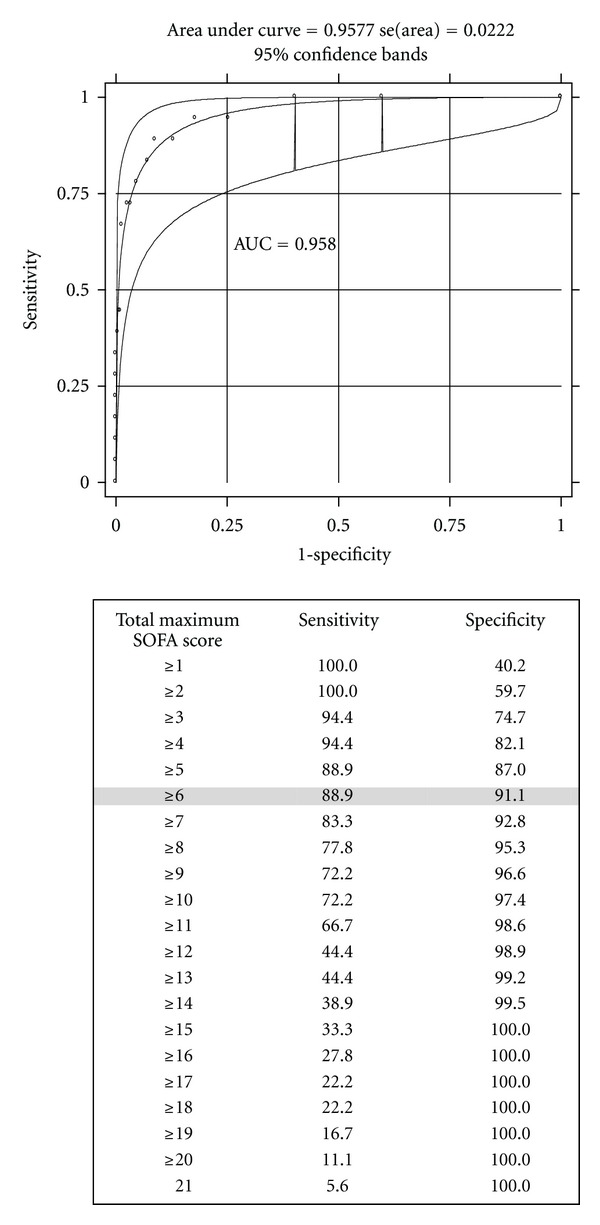
The area under the receiver operating characteristic (ROC) curve for total maximum SOFA (Sequential Organ Failure Assessment) score with different cutoff points as predictors of death among cases of severe maternal morbidity.

The performance of total maximum SOFA score is evaluated in Figure 3. Interpretation of the area under the ROC curve showed that the performance of total maximum SOFA score for the obstetrical population with SMM was excellent (AUC 0.958; 95% CI: 0.914–1.0). When the performance of maximum SOFA scores was calculated individually for each organ function, the discriminatory power of hepatic and neurological function scores was found to be poor. The best results were obtained, respectively, from the cardiovascular and respiratory scores; however, no individual score alone had better discriminatory power than the total maximum SOFA score (Table 3).

## 5. Discussion

The present study investigated organ dysfunction/failure in 673 cases of SMM admitted to an obstetric ICU over a five-year period. This study was the first carried out to evaluate the performance of total maximum SOFA score for cases of SMM, the results of which revealed the excellent performance of this score in this patient population.

The term SMM has been used to describe pregnant or recently delivered women with potentially life-threatening conditions and maternal near miss who survive [37]. Scores based on criteria of organ dysfunction/failure have greater discriminatory power in cases of SMM [8–10, 12, 14]. Therefore, a maternal life-threatening condition would be the women with organ dysfunction or failure [4].

The APACHE II and SAPS II, the scores most commonly used in obstetric populations [20], overestimate severity and maternal mortality ratios [8, 9, 12, 14, 19, 20]. The factors that would render application of this method difficult in this group of patients would be the physiological changes that occur during pregnancy and the transitory nature of obstetric morbidities [19, 30].

The decision to use the total maximum SOFA score in this study was made primarily in view of the performance of this score in different patient populations (AUC > 0.800) [10, 25, 27] and because the total maximum SOFA score uses simple variables that are easily standardized, easily measured without the need for high-complexity resources [24, 25, 27, 38]. The daily evaluation, although appealing, can miss the total amount of organ dysfunction sustained by the women, leading to an underestimation of the cumulative insult suffered [28].

The total maximum SOFA score permitted analysis of the entire pathophysiologic process of SMM, the aggregated score reflecting the maximum degree of alteration in organ function at different moments throughout the time the patient remained in the ICU. Among the possible motives for the better performance of total maximum SOFA score compared to each individual organ function score alone, emphasis should be given to the phenomena of interdependence of organ dysfunction, which are multiple and not always easily recognized [9, 11, 26, 28].

There are some limitations to this study that should be taken into consideration. First, it is a retrospective study in which data collection was performed manually by reviewing medical charts. Secondly, evaluation of the performance of scores may have been affected by the relatively few maternal deaths that occurred in the study (18 deaths). Third, the study was carried out in a tertiary referral hospital for cases of SMM, and the results obtained may not be completely applicable to other patient populations.

In accordance with studies carried out in clinical and surgical ICUs, the obstetrical complications in this population were directly responsible for the majority of admissions [9, 10, 20]. The maternal mortality rate of 2.6% in the ICU was within the range reported in recent studies in similar patient populations (2.3–27%) [8–10, 12, 14, 18, 19, 39].

Of the total number of admissions to the ICU, failure of one or more organs occurred in 20.5% of cases. Studies carried out in clinical/surgical ICUs have registered a proportionally greater number of cases of organ failure among cases of SMM admitted to their units (40–65%) [8, 9, 14]. This difference in the percentage of cases of organ failure is explained by the particular characteristics of an obstetric ICU where a considerable number of women are admitted for monitoring and surveillance prior to or following the resolution of pregnancy [35].

Organ failure was an integral part of the pathophysiologic process that led to death in almost all the cases of SMM. Of the 18 deaths, only one did not involve organ failure, at least identified by these means. In this specific case, a pregnant woman with advanced neoplastic disease received palliative care for the entire time required to achieve fetal pulmonary maturation and termination of the pregnancy. The evaluation of organ failure according to the maximum SOFA score was hampered because laboratory tests and/or advanced life support procedures and interventions were not carried out.

Higher maximum SOFA scores were associated with mortality, and this association was most evident in the hepatic and neurological scores. Nevertheless, when these two components were analyzed, their discriminatory power was found to be poor. The few cases of organ failure may have negatively affected evaluation of the performance of these scores; however, in previous studies, diagnosis of hepatic failure (maximum score ≥ 3) was also found not to contribute significantly towards the prediction of outcome of hospitalization [9, 28, 39]. With respect to the neurological score, use of the Glasgow coma scale may have affected its performance, since the number of neurological failures may have been underestimated due to the use of sedation in the more severe cases [26, 28, 38].

Cardiovascular and/or respiratory failure was significantly correlated with prognosis, and this finding was also reported by investigators who evaluated cases of SMM in ICUs [8, 9, 14] and in other populations of severely ill patients [28, 38]. The discriminatory power of SOFA score for coagulation was lower than these scores. The performance of this score may have been affected by the transitory nature of thrombocytopenia and its reversibility following resolution of the pregnancy and the obstetric complications. This result is similar to those found in studies carried out in clinical/surgical ICUs, where coagulation failure (≤50,000 platelets) did not significantly affect the prognosis of the patient [9, 28, 38].

## 6. Conclusions

This study showed the excellent performance of the total maximum SOFA score in cases of SMM admitted to an ICU. The total maximum SOFA score proved capable of evaluating the severity and prognosis of this patient population, and its discriminatory power appears not to have been affected by the physiological modifications of pregnancy. The evaluation of organ dysfunction/failure according to maximum SOFA score is simple, easily standardized, and requires only low-complexity laboratory resources. Use of the maximum SOFA score for the conceptual and operational definition of cases of SMM may contribute towards the efforts of healthcare institutions and organizations in identifying new auditing strategies to reduce maternal mortality worldwide. In fact, the preliminary results of the current study were presented in a meeting of the WHO working group on maternal mortality and morbidity which was held in Geneva, Switzerland in 2008, and they were taken into account for the WHO decision of using organ dysfunction/failure markers as its official criteria for maternal near miss [37, 40] which are being currently tested in the field.

## Figures and Tables

**Table 1:** Mean and standard deviation of maximum Sequential Organ Failure Assessment (SOFA) score for each organ or system evaluated and the total score, according to the outcome of cases of severe maternal morbidity admitted to an obstetric intensive care Unit.

Organ or system evaluated	Outcome		Total	*P* value*
	Death	Survivor		
Respiratory	2.61 (1.46)	0.41 (0.96)	0.47 (1.04)	<0.001
Coagulation	1.89 (1.45)	0.80 (1.05)	0.83 (1.07)	<0.002
Hepatic	1.28 (1.64)	0.14 (0.52)	0.17 (0.61)	<0.001
Cardiovascular	3.22 (1.56)	0.28 (0.87)	0.36 (1.01)	<0.001
Neurologic	1.78 (1.90)	0.07 (0.41)	0.12 (0.57)	<0.001
Renal	1.28 (1.60)	0.17 (0.67)	0.20 (0.73)	<0.001
Maximum total SOFA score	12.06 (5.47)	1.87 (2.56)	2.14 (3.14)	<0.001

Total	18	655	673	

*Mann-Whitney test.

**Table 2:** Estimated probability of death of women with SMM, for each value of the total maximum Sequential Organ Failure Assessment score and the corresponding adjusted logistic regression equation.

Total maximum SOFA score	Probability of death (%)
1	0.27
2	0.44
3	0.72
4	1.16
5	1.87
6	3.00
7	4.78
8	7.53
9	11.67
10	17.65
11	25.81
12	36.08
13	47.80
14	59.77
15	70.68
16	79.64
17	86.39
18	91.15
19	94.35
20	96.44
21	97.78
22	98.62
23	99.14
24	99.47
	
Adjusted logistic regression equation:	
Prob (death) = 1/(1 + exp(−(−6.380 + 0.484 × SOFA)))	

SOFA: Sequential Organ Failure Assessment.

**Table 3:** Values of sensitivity and specificity of individual maximum Sequential Organ Failure Assessment (SOFA) score of each system evaluated, for the prediction of maternal death, according to cutoff points of the score.

System evaluated	Sensitivity	Specificity	AUC [95% CI]
Respiratory			0.903 [0.827–0.979]
≥1	83.3	81.7	
≥2	83.3	86.3	
≥3	55.6	92.7	
4	38.9	98.2	
Coagulation			0.728 [0.588–0.869]
≥1	72.2	54.2	
≥2	61.1	76.0	
≥3	44.4	92.2	
4	11.1	97.6	
Hepatic			0.707 [0.431–0.983]
≥1	44.4	92.1	
≥2	38.9	95.6	
≥3	27.8	98.6	
4	16.7	99.8	
Cardiovascular			0.906 [0.747–1.0]
≥1	83.3	87.9	
≥2	83.3	92.7	
≥3	77.8	95.7	
4	77.8	96.0	
Neurologic			0.747 [0.438–1.0]
≥1	50.0	95.9	
≥2	50.0	98.2	
≥3	44.4	98.9	
4	33.3	99.7	
Renal			0.889 [0.797–0.981]
≥1	55.6	91.8	
≥2	27.8	96.3	
≥3	22.2	97.4	
4	22.2	97.9	

AUC: area under the curve.
